# Severe disseminated Nocardia brasiliensis pneumonia with normal immune function: A case report

**DOI:** 10.1097/MD.0000000000036402

**Published:** 2024-01-05

**Authors:** Xiaobin Wang, Hongjun Yang, Yunbo Xie, Xuejiao Xian

**Affiliations:** a Department of General Surgery, The First Affiliated Hospital of Kunming Medical University, Kunming City, Yunnan Province, China; b Department of general surgery, Chuxiong Yi Autonomous Prefecture People’s Hospital, Lucheng Town, Chuxiong City, Chuxiong Yi Autonomous Prefecture, Yunnan Province, China.

**Keywords:** diagnosis, Nocardia brasiliensis, treatment

## Abstract

**Rationale:**

Members of the genus Nocardia brasiliensis are Gram-positive, aerobic bacteria and exist ubiquitously in most environments. In recent years, the incidence of Nocardia brasiliensis has increased significantly and become a global concern. It may be predominantly caused pulmonary infections in immunocompromised hosts. Interestingly, however, we found that it can be present not only on immunocompromised hosts, but also to infect patients with a normal immune system.

**Patient concerns:**

We report a very rare case of a 49-year-old immunocompetent man with disseminated Nocardia brasensis pneumonia. He had a fever for 14 days (maximum temperature about 38°C) and a history of mass rupture.

**Diagnoses:**

Severe Disseminated Nocardia brasiliensis pneumonia with normal immune function.

**Interventions:**

No.

**Outcomes:**

The patient was finally diagnosed with Severe Disseminated Nocardia brasiliensis pneumonia and received compound sulfamethoxazole treatment for 4 months.

**Lessons subsections:**

Our report highlights when cold pus appears in soft tissues such as the lower limbs, neck, nose, scalp, etc, should prompt timely evaluation and biopsy for definitive diagnosis. Be alert to a normally immunocompetent, disseminated Nocardia brasiliensis infection. Early recognition and effective treatment are necessary conditions for successful results. This would allow for better disease prognostication while enabling physicians to develop more effective treatment strategies.

## 1. Introduction

On Columbia blood agar, Nocardia brasiliensis (N. brasiliensis) forms white, chalky colonies that are Gram-positive branching, aerobic bacteria.^[1]^ Living in soil (an excellent source of oxygen since plants produce oxygen) and eating dead or decaying plants, this organism thrives.This infection is usually acquired through ingestion, inoculation, or punctures by something contaminated with N. brasiliensis.^[2]^ Nocardiosis, a rare but potentially fatal infection, is caused by this microbe. By interfering with the function of T-cells in the host, Nocardia brasiliensis creates an immunosuppressive environment in the body.^[3]^ Skin lesions are often the first symptom of Nocardia brasiliensis infection after it enters the body through the lungs. Those who are immunocompromised, HIV-positive, bone marrow transplant recipients, or who have taken corticosteroids for a long time are most likely to contract an infection.^[1]^ Here we report a case of disseminated Brazilian Nocardia pneumonia with normal immune function of Chinese nationality.

## 2. Case report

**Figure 1.**
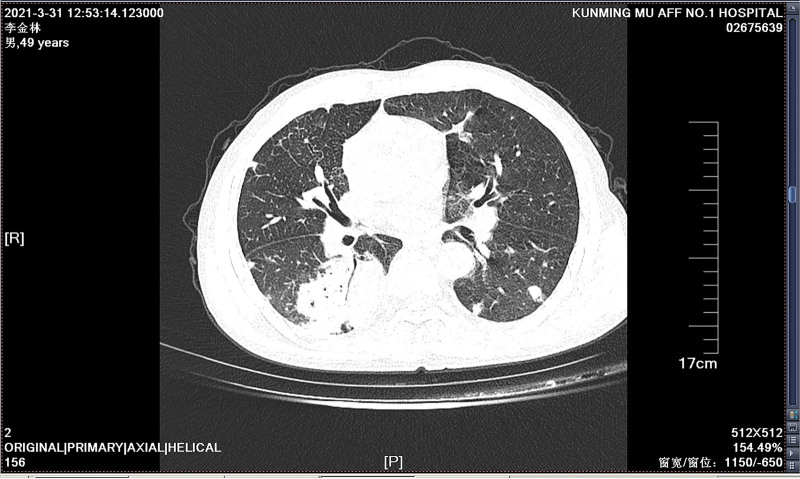
Chest CT on March 31, 2021 showed that large sheet consolidation of the upper and lower lobes of the right lung, bronchial air signs inside, multiple nodular high-density shadows in both lungs, and no definite enlarged lymph node shadows in the mediastinum. CT = computed tomography.

**Figure 2.**
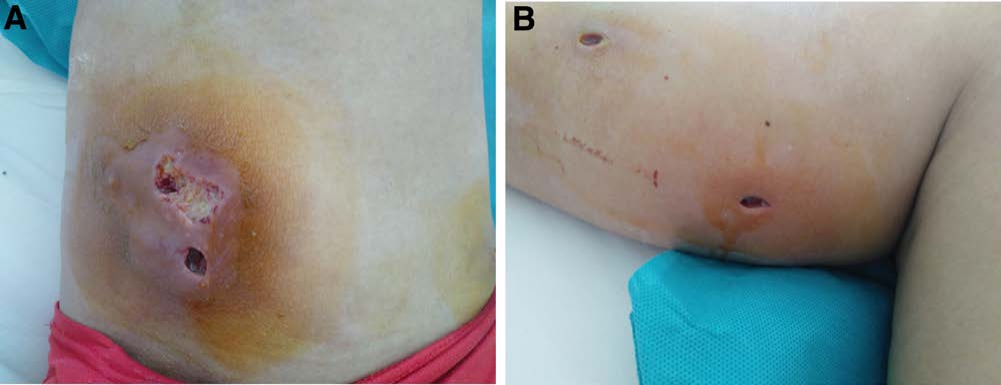
(A) Nocardia brasiliensis causes skin abscess. (B) Nocardia brasilianum causes skin abscess.

**Figure 3.**
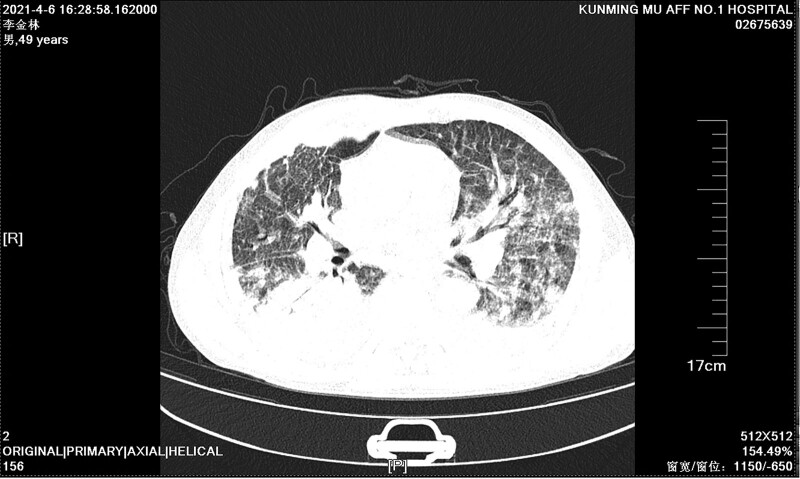
Chest CT on April 6, 2021 showed that the upper and lower lobes of the right lung had larger flaky consolidation, with bronchial air signs inside, multiple high-density nodules of varying sizes in both lungs, and thickened upper lobular lobules in both lungs. The exudation increased, and the mediastinum still has no clear enlarged lymph node shadow. CT = computed tomography.

**Figure 4.**
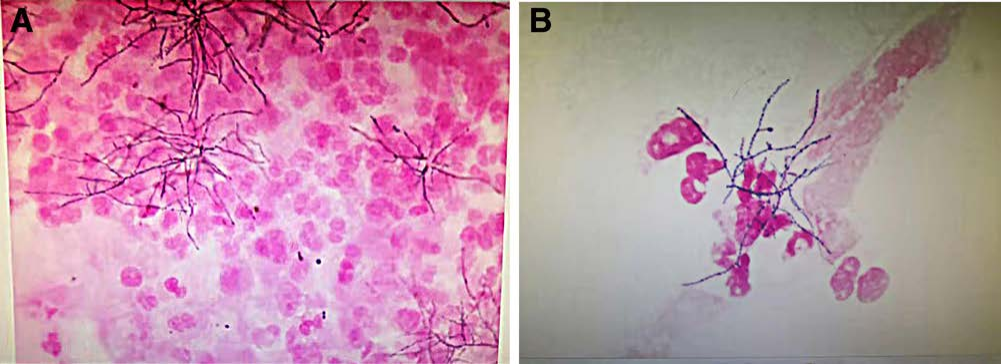
(A) Nocardia brasilianum HE stained smear. (B) Nocardia brasilianum HE stained smear.

**Figure 5.**
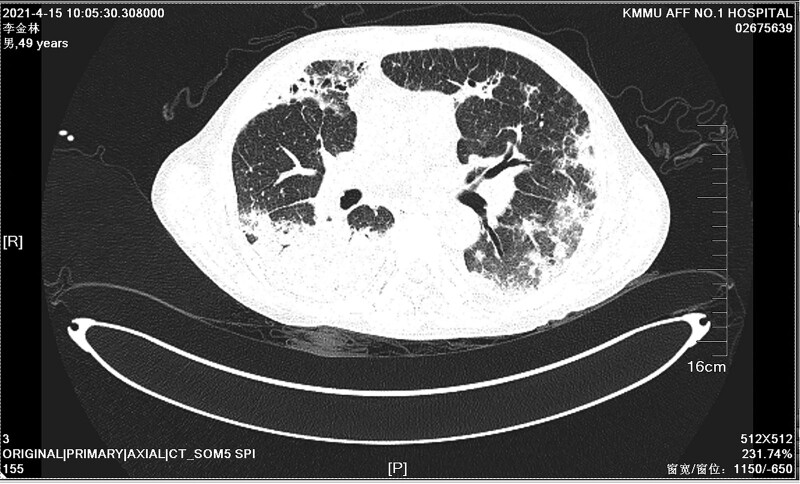
Chest CT on April 15, 2021 showed that the large flaky consolidation of the upper and lower lobes of the right lung was absorbed more than the previous part, and the bronchial air sign was seen inside, and the size of the lungs was multiple. The nodules vary from one to the other, some of them are smaller than before, the upper lobules of the lungs are thickened, and the exudation is absorbed more than before, and the mediastinum still has no clear enlargement of the lymph nodes. CT = computed tomography.

**Figure 6.**
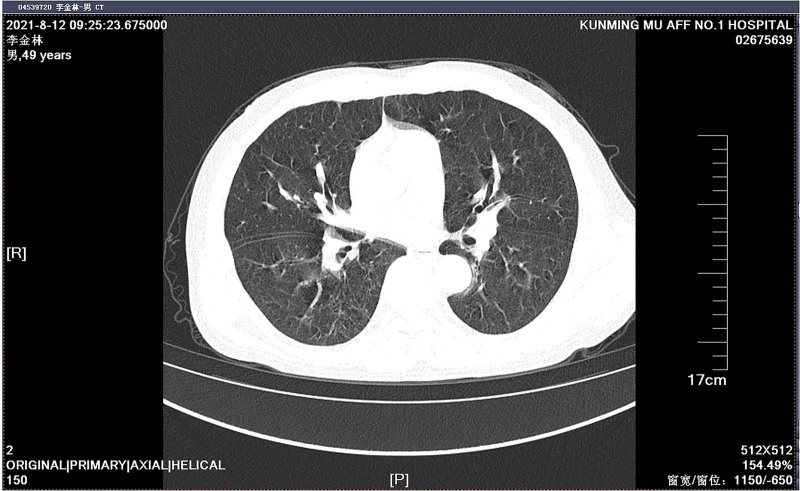
Chest CT on August 12, 2021 showed that multiple inflammatory consolidations in both lungs had been absorbed. CT = computed tomography.

**Figure 7.**
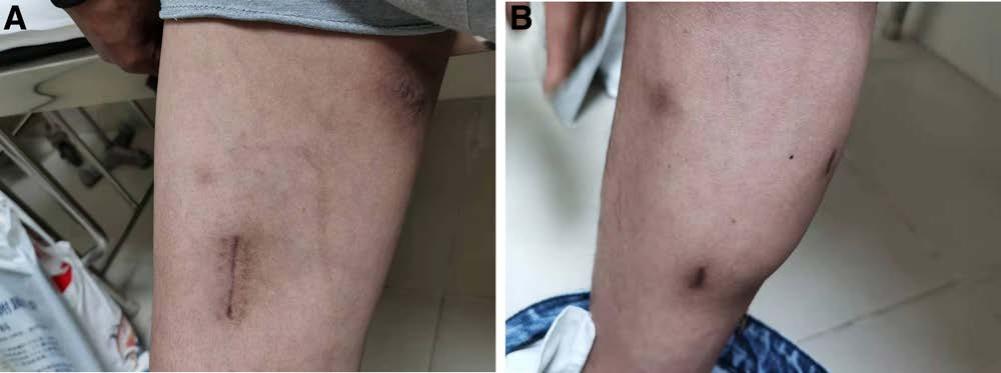
(A) Skin condition improves. (B) Skin condition improves.

A 49-year-old Chinese man working in a coal-fired power plant had a fever for 14 days (maximum body temperature was about 38°C) and had a history of ruptured lump. Denies the history of major diseases, denies long-term use of immunosuppressants and hormones. He was previously admitted to a primary hospital and received anti-infective treatment consisting of cefotaxime, tinidazole and levofloxacin. This series of anti-infective treatments did not improve his condition, so he was admitted to our hospital on March 31, 2021. Check the patient medical history, the patient has smoked for more than 20 years, and smoked about 10 cigarettes a day. There was no history of tuberculosis or trauma, positive tuberculin skin test, syphilis, gonorrhea, chlamydia, human immunodeficiency virus (HIV), malignancy, hepatitis, recent surgery or recent travel. On admission, the patient was generally in poor condition, clear of consciousness, poor mental state, fever, and multiple masses ruptured throughout the body; the first evaluation showed the body temperature was 39.2°, the blood pressure was 127/58 mm Hg, and the blood oxygen saturation level of 83%. The number of white blood cells of the patient on March 31, 2021 is 14.65*10^9/L (neutrophils: 87.80%; lymphocytes: 8.80%, eosinophils: 0.00%), The number of red blood cells is 3.07*10^12/L, and the number of platelets is 388*10^9/L. Chest CT showed: large sheet consolidation of the upper and lower lobes of the right lung, bronchial air signs inside, multiple nodular high-density shadows in both lungs, and no definite enlarged lymph node shadows in the mediastinum (Fig. 1). Distributed in the lower limbs, neck, nose, scalp and other soft tissue masses ulcerated and discharged pus, the masses without heat, redness, fever, significant pain, or inflammation, the pus is milky white mucus, nothing special odor. (Fig. 2A and 2B). Body temperature fluctuates (36.6°C~40.5°C), the heat type is atypical intermittent fever, and the peak is more common in the afternoon (between 14:00 and 18:00). Waiting for the results of the secretion culture of the ulcer, start piperacillintazole the broad-spectrum antibiotic treatment of batan 4.5 g q8h, metronidazole 0.5 g q8h and vancomycin 1.0 g q12h, combined with local abscess incision, vacuum sealing drainage vacuum sealing drainage of the wound, the patient still has high fever. 2021.04.06 chest CT showed that the upper and lower lobes of the right lung had larger flaky consolidation, with bronchial air signs inside, multiple high-density nodules of varying sizes in both lungs, and thickened upper lobular lobules in both lungs. The exudation increased, and the mediastinum still has no clear enlarged lymph node shadow (Fig. 3). On the 7th day, the secretion culture showed: Basinocardia (Fig. 4A and 4B), the patient immune function did not decrease significantly after reexamination, using imipenem and cilastatin sodium 1.0 g q6h, compound sulfamethoxazole two tablets of q12h antibiotic therapy, while supporting the breathing and vital organs, during which the peak body temperature decreased significantly. The patient respiratory tract alveolar lavage fluid smear on April 13 showed concurrent infection with drug-resistant Acinetobacter baumannii, according to the drug sensitivity test, it is only sensitive to tigecycline, and the use of tigecycline 50 mg q12h is increased on the basis of the original treatment. In the next few days, it was observed that the patient general condition gradually improved, and the chest CT was significantly improved: 2020.04.15, the large flaky consolidation of the upper and lower lobes of the right lung was absorbed more than the previous part, and the bronchial air sign was seen inside, and the size of the lungs was multiple The nodules vary from one to the other, some of them are smaller than before, The upper lung lobules are thickened, the exudate is absorbed more than before, and the mediastinum still has no obvious enlarged lymph nodes (Fig. 5). When she was discharged from the hospital on May 6, her temperature had dropped to normal. Discontinued imipenemcitatin sodium 1.0 g q6h and tigecycline 50 mg q12h, and continued to take compound sulfamethoxazole tablets 3 qid. The drug was discontinued on August 7, and the chest CT was reexamined on August 12, 2021. Multiple inflammatory consolidations in both lungs were absorbed (Fig. 6). The patient body abscess improved significantly (Fig. 7A and 7B).

## 3. Results

We drained the pus from soft tissue masses in the lower limbs, neck, nose, and scalp. The patient was also instructed to begin to receive compound sulfamethoxazole treatment for 4 months. When the patient no longer had a fever, we recheck the chest CT and it scan showed that Compared with the previous CT reports, multiple inflammatory consolidations in both lungs had been absorbed. No relapse occurred over the next 2 years of follow up.

## 4. Discussion

Nocardia is a gram-positive bacillus with the microscopic appearance of branched hyphae, which can produce quite serious diseases in a suitable host. Early recognition and effective treatment are necessary conditions for successful results. Although nocardiosis usually occurs in patients with cell-mediated immunosuppressive diseases, infections sometimes occur in patients with normal immune functions.^[1]^

Nocardia is a ubiquitous environmental organism that can cause local or disseminated infections in humans. Causes a variety of diseases, infects the lungs, skin, soft tissues, and in some cases also infects the central nervous system.^[2]^ Among them, the lung is the most common site of primary infection, and central nervous system infections are often encountered through blood spread in lung lesions, especially in hosts with weakened immune functions.^[1]^ This is because inhalation is the main source of exposure to this microorganism, and its symptoms include: cough, shortness of breath, chest pain, hemoptysis, fever, night sweats, weight loss and fatigue. But skin damage may still be the first symptom of infection. Primary skin and soft tissue nocardiac disease can occur due to any type of skin damage, including soil contamination, superficial abscesses, or local cellulitis that may appear on the skin.^[3,4]^ After repeated medical history inquiries, the patient still denied the history of trauma, but strangely, the patient had no obvious respiratory symptoms such as cough, shortness of breath, chest pain, hemoptysis, etc. In addition, this patient is an extremely rare patient with Basinocardium lung infection with normal immune function. He had not previously received a bone marrow transplant and denied long-term use of glucocorticoids and immunosuppressive drugs. Laboratory test: HIV screening was negative. This reminds us that even if the patient does not have obvious respiratory symptoms such as cough, shortness of breath, chest pain, hemoptysis, etc, the lung infection of Nocardia brasiliensis cannot be easily ruled out.

Analysis of the patient lymphocyte subsets showed that the proportion and absolute value of T lymphocytes were normal (CD3+/CD45 + was 77.87%, and CD3+/CD45+# was 1232 cells/µL), while the proportion and absolute value of B lymphocytes decreased (CD3 -CD19+/CD45 + is 3.13%, CD3-CD19+/CD45+# is 49/µL). Some studies have shown that the host protective immune response to N. brasiliensis involves cell-mediated immunity.^[5,6]^ In the early stage of infection, local polymorphonuclear cells infiltrate, and then phagocytes and monocytes infiltrate the site of infection.^[3]^ N. brasiliensis is an intracellular bacteria that can survive and reproduce in macrophages.^[7]^ We know very little about how N. brasiliensis evades the microbicidal mechanism developed by the host.

Trimethoprim-sulfamethoxazole is still the drug of choice for initial treatment, because Nocardia is often sensitive to this drug, and it has been the cornerstone of nocardiosis treatment for many years.^[5,8,9]^ Therefore, we used trimethoprim-sulfamethoxazole for the first time when confirming that the patient was infected with Brazil Nocardia. Patients with lung or multifocal (non-central nervous system) nocardiac disease with normal immune function can achieve curative effect through 6 to 12 months of antibacterial therapy.^[1]^ However, it is worth noting that when using compound sulfamethoxazole tablets, because the drug has the effect of inhibiting platelets, it is necessary to pay attention to monitoring liver and kidney function, platelets, red blood cells, hemoglobin, etc. Currently, there is no unified treatment method for Nocardia infections due to differences in pathogenicity and antibiotic susceptibility among Nocardia species. Treatment of Nocardia infections with sulfonamides (such as trimethoprim and sulfamethoxazole) is considered to be most effective. However, there are many reports of patients showing resistance to these drugs.^[6,10]^ Imipenem, ceftriaxone, linezolid, levofloxacin, amikacin, amoxicillin and clavulanate potassium, clarithromycin and cefotaxime are some of the alternatives to sulfa drugs or in combination with sulfa drugs Drugs,^[11]^ and can reduce the resistance of xanthamine drugs. Clinical treatment depends on the patient drug sensitivity and the severity of the disease.

Another noteworthy problem is that the patient skin nodules are cold abscesses, without fever, redness, fever, obvious pain or inflammation. This finding is similar to that of Li Siying et al.^[12]^ And Alison M. Fernandes et al^[13]^ Consistent with the findings of Ana María Medina-Torres et al^[14]^ This suggests that when cold pus appears in soft tissues such as the lower limbs, neck, nose, scalp, etc., Basinocardium infection cannot be ruled out.

## 5. Study limitations

The method we use to diagnose Nocardia brasiliensis infections is a combination of laboratory tests and cultures of secretion from ruptured abscesses, which is less precise and faster than molecular methods, such as 16S rRNA, hsp65, and secA1 gene sequencing, it makes us significantly late in the medications for Nocardia brasiliensis. Had it not been for the fact that the patient was young and not immunodeficient, and the extensive clinical experience of the team of doctors that allowed us to respond quickly to the symptoms that arose, there is a possibility of a very bad clinical outcome without waiting for the results of the culture of the secretions. But it is worth noting that secretion cultures in combination with a laboratory test do not require a lot of equipment, and the diagnosis can be made even in primary hospitals.

## 6. Conclusions

When cold pus appears in soft tissues such as the lower limbs, neck, nose, scalp, etc, should prompt timely evaluation and biopsy for definitive diagnosis, particularly in immunocompromised patients, including people with HIV. Although nocardiosis usually occurs in patients with cell-mediated immunosuppressive diseases, infections sometimes occur in patients with normal immune functions. Early recognition and effective treatment are necessary conditions for successful results.

## Author contributions

**Data curation:** Xiaobin Wang, Hongjun Yang.

**Investigation:** Hongjun Yang.

**Methodology:** Hongjun Yang, Yunbo Xie.

**Supervision:** Xuejiao Xian.

**Validation:** Yunbo Xie, Xuejiao Xian.

**Visualization:** Xuejiao Xian.

**Writing – original draft:** Xuejiao Xian, Xiaobin Wang.

**Writing – review & editing:** Xuejiao Xian.
